# CONSISTENCY IN DOCUMENTATION OF TRIGGER EVENTS AND RELATED DIAGNOSES DURING 911 TRANSFERS TO ED NH RESIDENTS

**DOI:** 10.1093/geroni/igz038.1884

**Published:** 2019-11-08

**Authors:** Colin Reid, University of British Columbia, Kelowna, British Columbia, Canada Chloe Sollid-Gagner, Rachel Ma, Lee Morgan, Kaitlyn C Tate, Greta G Cummings

**Affiliations:** 1 University of British Columbia, Kelowna, British Columbia, Canada; 2 University of Alberta, Edmonton, Alberta, Canada

## Abstract

Previous research has identified that documentation practices during resident transitions from long-term care (LTC) to the emergency department (ED) can be inconsistent or nonspecific, leading to the receipt of inappropriate or insufficient care. When many older adults (>65 years) in LTC have cognitive impairments that make communication difficult and changes in resident health can present ambiguously, consistent documentation becomes particularly critical for the provision of safe, timely and appropriate care. The purpose of this study was to examine documentation practices across care settings related to reason for transfer during transitions of older adults from LTC to the ED. We tracked every resident transfer from 38 participating LTC facilities to two included EDs in two Western Canadian provinces from July 2011 to July 2012. Using case-related data gathered from 637 transitions, we employed qualitative content analysis to categorize whether documentation practices from LTC to the ED were sufficiently reported and clinically consistent. Transitions were defined as consistent when symptomatology, trigger events and diagnoses aligned in a medically-intuitive manner. Inconsistency patterns were further categorized as minor (indicating one outlying symptom/trigger) and major (indicating more than two inconsistencies). Of the total 637 transitions, 2.67% contained too little data to be accurately categorized. The majority of cases (75.82%) of cases had consistent documentation, 13.19% had minor inconsistencies, and 8.32% had major inconsistencies. These results support that shared continuing education for documentation practices should occur across care settings to ensure that documentation practices are sufficient, support a geriatric focus and consider differing clinician perspectives.

